# Real World Study on the Best CPX‐351 Treatment Duration and Timing for Allogeneic Stem Cell Transplantation

**DOI:** 10.1002/ajh.70083

**Published:** 2025-09-24

**Authors:** Fabio Guolo, Luana Fianchi, Maria Paola Martelli, Federico Lussana, Francesco Grimaldi, Federica Pilo, Michela Rondoni, Carla Filì, Paola Minetto, Debora Capelli, Patrizia Chiusolo, Massimo Breccia, Sara Mastaglio, Massimo Bernardi, Monica Bocchia, Monica Fumagalli, Sara Galimberti, Valentina Mancini, Anna Lisa Piccioni, Luca Maurillo, Nicola Stefano Fracchiolla, Raffaele Palmieri, Calogero Vetro, Cristina Papayannidis, Lorenzo Brunetti, Alessandra Sperotto, Federica Gigli, Patrizia Zappasodi, Antonino Mulé, Caterina Patti, Erika Borlenghi, Michelina Dargenio, Federica Lessi, Marco Cerrano, Daniela Cilloni, Alessandro Isidori, Monia Lunghi, Caterina Alati, Carmela Gurrieri, Carola Riva, Giovanni Marconi, Ivana Lotesoriere, Samuele Gatani, Anna Maria Scattolin, Manuela Caizzi, Salvatore Perrone, Atto Billio, Filippo Gherlinzoni, Francesco Mannelli, Michele Gottardi, Roberto Cairoli, Anna Candoni, Felicetto Ferrara, Livio Pagano, Roberto Massimo Lemoli, Adriano Venditti, Elisabetta Todisco

**Affiliations:** ^1^ University of Genoa Department of Internal Medicine (DiMI) Genoa Italy; ^2^ IRCCS Ospedale Policlinico San Martino Dipartimento di Oncologia ed Ematologia Genoa Italy; ^3^ Fondazione Policlinico Universitario “A. Gemelli” IRCCS, UOC Ematologia e TCSE Rome Italy; ^4^ Università di Perugia, Ospedale “Santa Maria della Misericordia” Dipartimento di Medicina e Chirurgia Perugia Italy; ^5^ ASST Papa Giovanni XXIII, Hematology and Bone Marrow Transplant Unit Bergamo Italy; ^6^ Università degli Studi di Napoli “Federico II” Naples Italy; ^7^ Ospedale Oncologico di Riferimento Regionale “Armando Businco”, Hematology and Bone Marrow Transplantation Unit Cagliari Italy; ^8^ Hematology Unit and Metropolitan Romagna Transplant Network Ravenna Italy; ^9^ Azienda Sanitaria Universitaria Friuli Centrale (ASUFC) Division of Hematology and Stem Cell Transplantation Udine Italy; ^10^ Azienda Ospedaliera Universitaria Ospedali Riuniti di Ancona, Hematology Department University of Ancona Ancona Italy; ^11^ Universita' Cattolica del Sacro Cuore Department of Hematology Rome Italy; ^12^ Sapienza University Department of Translational and Precision Medicine Rome Italy; ^13^ Istituto di Ricovero e Cura a Carattere Scientifico (IRCCS) san Raffaele Scientific Institute Department of Onco‐Hematology Milan Italy; ^14^ Azienda Ospedaliera Universitaria Senese, Hematology Unit Siena Italy; ^15^ Ospedale “San Gerardo”, ASST di Monza, SC Ematologia Monza Italy; ^16^ University of Pisa, Department of Clinical and Experimental Medicine, Section of Hematology Pisa Italy; ^17^ ASST Grande Ospedale Metropolitano Niguarda, Niguarda Cancer Center Department of Hematology Milan Italy; ^18^ San Giovanni Hospital, Hematology Rome Italy; ^19^ University Tor Vergata Department of Biomedicine and Prevention, Hematology Rome Italy; ^20^ Fondazione IRCCS ca' Granda‐Ospedale Maggiore Policlinico, UOC Ematologia Milan Italy; ^21^ AOU Policlinico “G. Rodolico‐San Marco”, Divisione di Ematologia Catania Italy; ^22^ IRCCS Azienda Ospedaliero‐Universitaria di Bologna, Istituto di Ematologia “Seràgnoli” Bologna Italy; ^23^ Veneto Institute of Oncology IOV‐IRCCS, Onco Hematology Department of Oncology Padua Italy; ^24^ IRCCS Istituto Europeo di Oncologia, Divisione di Oncoematologia Milan Italy; ^25^ Fondazione IRCCS Policlinico “San Matteo” Dipartimento di Oncoematologia Pavia Italy; ^26^ Azienda Ospedaliera Riunita (AOR) Villa Sofia‐Vincenzo Cervello, Onco‐Hematology Unit Palermo Italy; ^27^ AO Spedali Civili, Operational Unit of Hematology Brescia Italy; ^28^ Ospedale Vito Fazzi Lecce Italy; ^29^ University of Padua Department of Medicine, Hematology and Clinical Immunology Unit Padua Italy; ^30^ AOU Città della Salute e della Scienza, Torino, Divisione di Ematologia Dipartimento di Oncologia Turin Italy; ^31^ AO Ordine Mauriziano, SCDU Ematologia e Terapie Cellulari Turin Italy; ^32^ AORMN Hospital, Hematology and Stem Cell Transplant Center Pesaro Italy; ^33^ AOU “Maggiore della Carità”, SCDU Ematologia Novara Italy; ^34^ “Bianchi‐Melacrino‐Morelli” Hospital, Hematology Unit, Hemato‐Oncology and Radiotherapy Department Reggio Calabria Italy; ^35^ ASST Valle Olona, Ospedale Di Busto Arsizio Varese Italy; ^36^ Unità Operativa di Ematologia, Ospedale dell'Angelo Venezia Italy; ^37^ S.C. Ematologia, Azienda Sanitaria Universitaria Integrata di Trieste Trieste Italy; ^38^ Department of Hematology Polo Universitario Pontino, S.M. Goretti Hospital Latina Italy; ^39^ Ospedale di Bolzano Bolzano Italy; ^40^ Ospedale ca' Foncello Treviso Italy; ^41^ Azienda Ospedaliero‐Universitaria Careggi Hematology Department Florence Italy; ^42^ Università degli Studi di Modena e Reggio Emilia Modena Italy; ^43^ AORN “A. Cardarelli”, UOC Ematologia Naples Italy

**Keywords:** acute myeloid leukemia, allogeneic stem cell transplantation, CPX‐351, drug optimization

## Abstract

In the registration clinical trial 301 (NCT01696084), CPX‐351 has shown to be superior to conventional 3 + 7 in secondary AML (s‐AML). However, the optimal duration of treatment, the best timing for allogeneic stem cell transplantation (allo‐HSCT), and the activity of CPX‐351 in specific s‐AML subgroups are unclear. To evaluate these aspects, a total of 513 s‐AML patients (median age 65.6 years, 19–79) treated with CPX‐351 were retrospectively analyzed. Complete remission (CR) rate after induction was 297/513 (58%), increasing to 340/513 (66%) after cycle 2. Among the 340 responding patients, 118 (34.7%), 137 (40.3%), and 85 (25%) received none, one, or two consolidation cycles of CPX‐351, respectively. Overall, 230/513 patients (48.8%) received allo‐HSCT. Median follow up was 23.66 months and median overall survival (OS) was 16.23 months. Patients with mutated *NPM*1 or with ELN 2017 favorable risk (*p* < 0.05) had a significantly longer OS (*p* < 0.05). In a landmark analysis, receiving allo‐HSCT was associated with a longer survival (Median OS not reached vs. 16.3 months for patients receiving or not receiving allo‐HSCT, *p* < 0.05). Completion of all allowed CPX‐351 cycles was beneficial only in patients not proceeding to transplant (*p* < 0.05), whereas in transplanted patients additional CPX‐351 cycles did not improve outcome. Our analysis suggests that also s‐AML patients with *NPM1* mutations and those belonging to the ELN 2017 favorable risk category benefit from CPX‐351. In eligible patients, allo‐HSCT should be performed as soon as a CR is achieved, whereas patients not undergoing transplant benefit from a complete CPX‐351 schedule.

## Graphical abstract

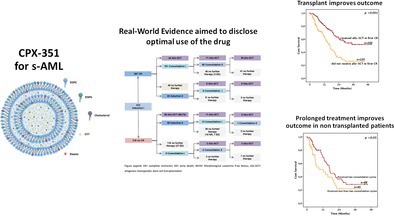

## 1 Introduction

Patients with secondary Acute Myeloid Leukemia (s‐AML) arising from a previous myelodysplastic syndrome (MRC‐AML) or secondary to chemotherapy (t‐AML) have a worse prognosis if compared to patients with “de novo” AML [1, 2, 3, 4, 5]. The dismal outcome of this subgroup of patients is explained by several reasons. They are usually older and have more frequently have high‐risk features such as unfavorable cytogenetics and molecular profile [2, 3, 4, 5]. Moreover, in patients with t‐AML, the toxicity of induction chemotherapy is magnified due to the previous exposure to cytotoxic chemotherapy for unrelated neoplasms [2, 3, 4, 5, 6]. As a result, most s‐AML patients respond poorly to conventional chemotherapy [3]. In this setting, the main curative option for eligible patients is allogeneic stem cell transplantation (allo‐HSCT), which is, however, more effective if a complete remission (CR) is achieved before transplant. Conversely, the goal of a durable CR is significantly less accomplished in s‐AML patients receiving only conventional treatment with no following allo‐HSCT [2, 3, 4, 5, 6].

The positive results from the Phase III 301 trial (ClinicalTrials.gov Identifier: NCT01696084), demonstrating the superiority of CPX‐351 over “3 + 7” in terms of CR rate and survival, ended in its approval by both FDA and EMA for the treatment of s‐AML, as defined per the former WHO 2016 classification [7].

Indeed, with a significantly longer follow‐up of 5 years, the survival advantage of CPX‐351 was confirmed in the long term, both in patients proceeding to allo‐HSCT or not. At the same time, Real‐World Evidences (RWE) from the USA, Italy, France, Germany, and the United Kingdom were generated, corroborating the efficacy and the tolerability of the CPX‐351 [7, 8, 9, 10, 11, 12, 13, 14, 15].

The role of RWE is critical to investigate mechanisms explaining the superiority of CPX‐351 over 3 + 7, which were not investigated in the phase III 301 trial. Indeed, RWE suggests that CPX‐351 may induce more frequently a negative minimal residual disease CR status [9, 10, 11, 12]. This observation is supported by a recent retrospective analysis confirming a higher probability of achieving MRD negativity with CPX‐351 in s‐AML patients if compared to conventional intensive chemotherapy [16]. Another explanation may be that CPX‐351 is significantly better tolerated than 3 + 7, mostly due to less severe extra‐hematological toxicity. In experimental models, it was also demonstrated that CPX‐351 does not damage the gastro‐intestinal mucosa by promoting eubiosis, opposite to “3 + 7” that causes dysbiosis. Such a protective mechanism prevents intestinal bacterial translocations into the blood stream, potentially resulting in less frequent severe bacteremias [17]. This observation is supported by recent data from the Italian SEIFEM group that confirm that, despite the frequency of bacteremia observed with CPX‐351 not being inferior to conventional chemotherapy, severe colitis and, consequently, severe sepsis caused by enteral bacteria are significantly less likely to occur in CPX‐351 treated patients [18]. Notwithstanding these favorable peculiarities, it is still unclear what the optimal number of CPX‐351 courses to deliver is or what the best timing is for performing allo‐HSCT after CPX‐351 induction [7, 8, 9, 10].

Recently, a British trial showed that CPX‐351 was superior to FLAG‐Ida chemotherapy (Fludarabine, High dose Cytarabine, and Idarubicin; FLAG‐Ida) in AML patients with secondary‐type mutations, whereas results were superimposable in the whole population, thus suggesting an increased activity in molecularly defined AML subtypes [18]. However, there is still scarce data on the efficacy of CPX‐351 in *NPM1* or *FLT3‐*ITD mutated AML or in patients with low‐risk AML, according to European Leukemia Net (ELN 2017‐2022) classification, as those subgroups are quite rare among s‐AML, therefore requiring a large patients' cohort in order to draw any conclusion. Furthermore, the efficacy of CPX‐351 in *TP53* mutated AML is still unclear [10, 12, 19].

Based on these premises, we analyzed the outcomes of a large cohort of 513 Italian patients who received on‐label CPX‐351 treatment since its approval, in order to evaluate the optimal duration of CPX‐351 treatment, the best timing for allo‐HSCT, and the efficacy of CPX‐351 on rare secondary AML subtypes such as those with *NPM1* or *FLT3*‐ITD mutations.

## 2 Methods

### 2.1 Study Design, Treatment and Diagnostic Procedures

This was a large multicenter retrospective study including patients affected by s‐AML according to WHO 2016 criteria, who received commercially available treatment with CPX‐351 in Italy between January 2019 and January 2022 in 38 Italian Centers.

As per label, all patients were allowed to receive up to two induction cycles with CPX‐351 and up to two consolidation cycles. CPX‐351 was administered at 44 mg/sqm (equivalent dose of Daunorubicin) on days 1, 3, and 5 on Induction I, on days 1 and 3 on Induction II, and at 29 mg/sqm on days 1 and 3 of each consolidation cycle.

No patients received any concomitant targeted anti‐leukemic drug during CPX‐351 treatment, or any maintenance treatment after achieving a complete remission.

Eligible patients proceeded to allo‐HSCT consolidation as per internal standard of each Center, and allo‐HSCT was allowed at any time during treatment, according to treating physician's judgment. Diagnostic workup was performed as per internal standard in all patients and included, in all patients, cytogenetic analysis and assessment of mutational status of *NPM1*, *FLT3*‐ITD. *TP53*, *RUNX1*, and *ASXL1* mutations were analyzed in most patients.

WHO 2016 classification was applied since it was adopted in order to evaluate eligibility to CPX‐351 treatment, whereas leukemia risk score was graded according to European LeukemiaNet (ELN) 2017 classification, since it was in use at the time of patient treatment and only a minority of patients had NGS data allowing for classification according to ELN 2022 risk score (Supporting Information) [20, 21, 22].

Cytogenetic‐molecular risk and treatment responses were defined according to the recommendations of the European LeukemiaNet 2017 [21]. Specifically, complete remission (CR), complete remission with incomplete recovery (CRi), morphological leukemia‐free state (MLFS), partial remission (PR), and non‐response or treatment failure (NR/SD/PD) were defined based on peripheral blood count and bone marrow blast percentage; timepoints for response assessment were not standardized and were defined upon investigator judgment.

The outcomes for effectiveness were the complete remission rate (CR), the overall survival (OS) defined as the time in months from the 1st day of treatment to death from any cause, and the event‐free survival (EFS) defined as the time in months from the 1st day of treatment to treatment failure, hematologic relapse from CR/CRh/CRi or death from any cause, whichever occurs first [21, 22].

CR was defined as an absolute neutrophil count of more than 1000 cells per cubic millimeter, a platelet count of more than 100,000/mm^3^, red‐cell transfusion independence, and bone marrow with less than 5% blasts. CRi was defined as all the criteria for CR, except for neutropenia (absolute neutrophil count, ≤ 500/mm^3^ or thrombocytopenia platelet count, ≤ 100,000/mm^3^) [21, 22].

### 2.2 Ethics Statement

The study was conducted in accordance with the Declaration of Helsinki and the Good Clinical Practice guidelines of the International Council for Harmonization. The protocol and related documents were approved by the Ethics Committee of the coordinating center on 06‐03‐2022 (n.606/23/06/2022) and subsequently by the ethics committee of each participating institution. This trial was registered on ClinicalTrial.gov. All authors had access to primary data.

## 3 Statistical Analysis

All analysis excluding landmark models were performed with IBM SPSS v22 running on a Linux environment. R statistical package (www.rproject.com) was used for landmark models and competing risk analysis. Different landmarks were considered when evaluating the impact of further CPX‐351 treatment delivered after the achievement of a complete remission, including patients alive and in CR at day 90, 120, and 150, in order to reduce the bias against patients receiving shorter treatment. In particular, day 90 was chosen as all responding patients achieved CR before day 90 (the last patient at day 88), and day 150 was chosen as all patients completed CPX‐351 treatment before day 150 (the last patient at day 147).

Dichotomous variables were compared using the Chi Square Test, or, where necessary, with the Fisher's exact Test. Continuous variables were compared with the Student's *T* Test, or, if normal distribution could not be confirmed, with Wilcoxon's Rank Test. A logistic regression analysis was performed for multivariate CR analysis [23].

Survival curves were built according to Kaplan–Meier's method, and univariate survival analysis was performed with the log‐rank test. A Cox proportional hazard model was built for each multivariate survival analysis, including only variables that fulfilled the proportional risk criteria. Transplant was considered as a time‐dependent variable [23].

All two‐tailed *p*‐value < 0.05 was considered statistically significant [23].

## 4 Results

### 4.1 Patients

513 s‐AML patients (264 male (51.5%) and 249 female patients (48.5%), median age 65.6 years, range 19–79) who received CPX‐351 treatment in 38 Italian Centers between January 2019 and January 2023 were included in this study. Eligible patients proceeded to allo‐HSCT consolidation as per the internal standard of each Center.

Of 513 patients, 108 (21.1%) and 405 (78.9%) were diagnosed with t‐AML or AML‐MRC, respectively.

Among the 405 patients with AML‐MRC, 101 (25%) received CPX based on the morphological criteria alone and 304 (75%) for a formal diagnosis of a previous MDS syndrome (including a previous diagnosis of Chronic Myelomonocytic Leukemia, CMMoL, in 15 patients) or the presence of MDS‐defining cytogenetics, with 84 of these 304 (27.6%) being previously treated with hypomethylating agents (HMA), for a median of 4 cycles (range 1–26 cycles).

Conventional cytogenetic analysis was performed in all patients. The majority of them carried an abnormal karyotype (357/513, 70%), mostly a high‐risk karyotype (297/357, 83%). Favorable‐risk cytogenetics was found in 5 t‐AML patients [t(8;21) in 3 and inv(16) in 2].

All patients were tested for *NPM1* and *FLT3‐*ITD mutational status. *NPM1* mutation was found in 31 patients (6%, 24 of them had t‐AML and 7 had MRC‐AML with a documented previous history of MDS); all patients with NPM1 mutations fulfilled criteria for CPX treatment (e.g., a history of MDS or additional molecular or cytogenetic changes characteristic of s‐AML). *FLT3*‐ITD mutation was present in 24 patients (4.6%). No patient had concomitant *FLT3*‐ITD and *NPM1* mutations. *TP53* was evaluated in 335 patients (65%), and mutations were found in 49 (15%): 12 t‐AML and 37 MRC‐AML. In 33/49 (67%) patients (8 t‐AML and 25 MRC‐AML), *TP53* mutation was associated with complex karyotype (CK, 9% of 335 patients evaluated for *TP53* mutations).

ELN 2017 score was favorable, intermediate, or high in 27 (5.2%), 177 (34.5%), and 309 (60.3%) patients, respectively. The 9 patients with *NPM1* mutations not belonging to the favorable ELN risk group had concomitant high‐risk cytogenetic features (hyperdiploid complex karyotype in 6, deletion of chromosome 7 in 2, and deletion of chromosome 5q in 1).

Most patients (84%) had at least one comorbidity, mainly cardiovascular diseases (43%) and type II diabetes (39%). Ninety‐seven (19%) of patients were aged more than 70 years. Patients' characteristics are summarized in Table 1.

**TABLE 1:** Patients characteristics.

		*N*. (%)
Total patients		513
Age (Median 65.6)	< 60 years	199 (38.8)
	60–70 years	217 (42.3)
	> 70 years	97 (18.9)
Karyotype	Low Risk	5 (1)
	Intermediate	211 (41.1)
	High Risk	297 (57.9)
*NPM1*	Mutated	31 (6)
	Wild Type	482 (93.9)
*FLT3‐ITD*	Positive	24 (4.7)
	Negative	489 (95.3)
*TP53*	Mutated	49/335 (14.6)
	Wildtype	286/335 (85.4)
ELN 2017	Favorable	27 (5.2)
	Intermediate	177 (34.5)
	Unfavorable	309 (60.3)
WHO 2016	MRC‐AML Morphology only	289 (56.3)
	MRC‐AML Previous MDS or MDS related cytogenetics	101 (19.7)
	MRC‐AML Previous CMMoL	15 (2.9)
	t‐AML	108 (21.1)
Previous HMA for MDS	Yes	84 (16.4)
	No	429 (83.6)
Bone marrow blasts	20%–30%	236 (46%)
	> 30%	277 (54%)

Abbreviations: CMMoL = chronic myelomonocitic leukemia; ELN = European LeukemiaNet; HMA = hypomethylating agents; MDS = myelodisplastic syndrome; WHO = World Health Organization.

### 4.2 Treatment Outline and Response to Treatment

**FIGURE 1:**
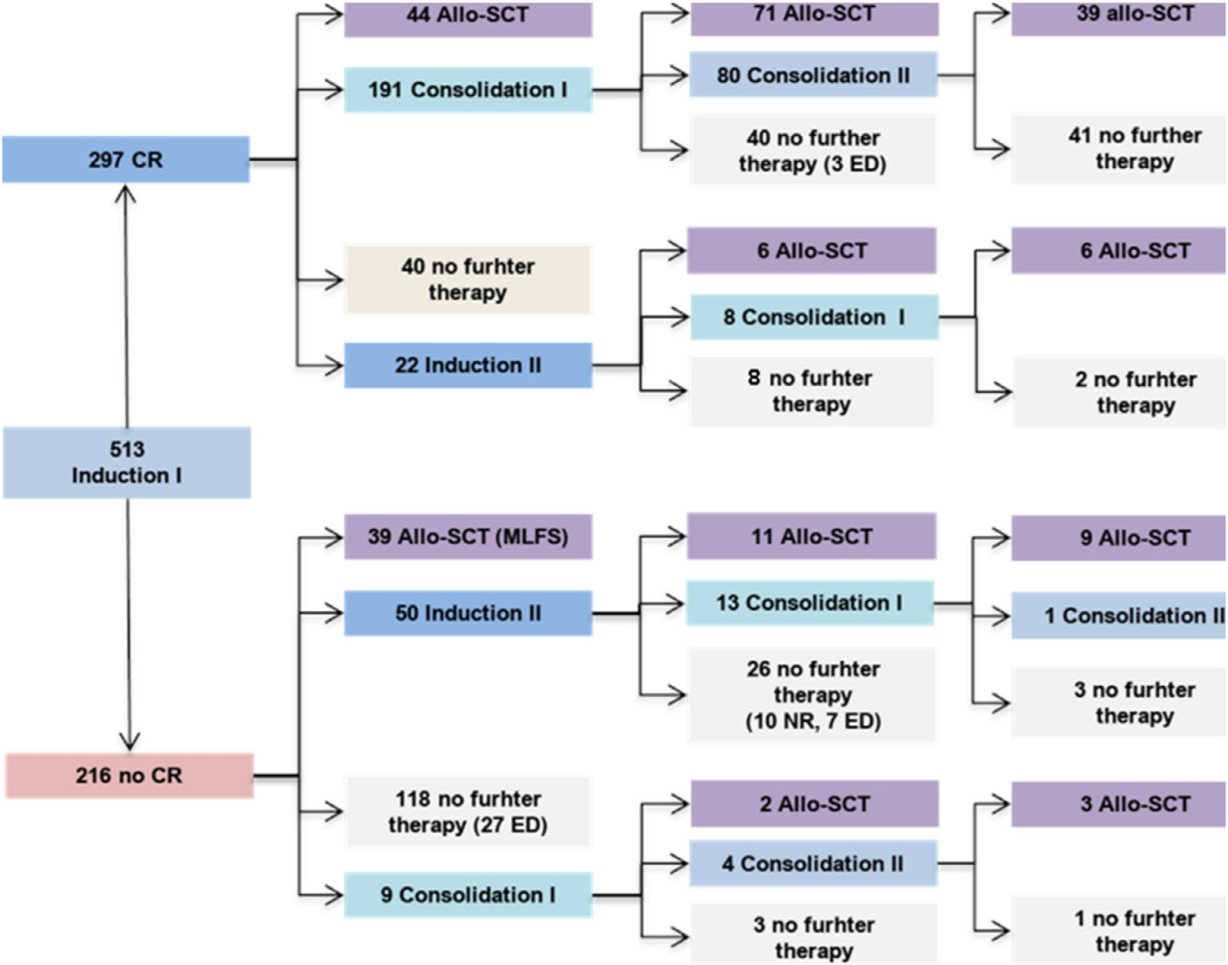
Treatment outline. Figure 1 shows the detailed sequence of CPX‐351 treatment administered to the 513 patients in the study, as well as the timing for allogeneic stem cell transplantation. [Color figure can be viewed at wileyonlinelibrary.com]

A detailed treatment overview is provided in Figure 1. After induction I, 297/513 patients (58%) achieved a complete remission (CR). Fifty patients received a second induction because of non‐response (*n* = 30) or partial remission (*n* = 20), and at this stage, 34 of them achieved a CR. The CR rate after II induction was 18/30 among NR patients (60%) and 16/20 among PR patients (80%). Nine PR patients received a consolidation cycle as a second course; all of them achieved a CR. Therefore, the CR rate increased to 66.3% after cycle 2 (340/513). Sixty‐two patients achieved morphological leukemia‐free status (MLFS) after cycle 1; 39 of them proceeded directly to allo‐HSCT, whereas the remaining 23 did not receive further CPX‐351 treatment. Consequently, a total of 363/513 (70.7%) patients achieved at least a morphological leukemia‐free status (MLFS) after cycle 2. Thirty‐ and 60‐day mortality were 5.2% and 8.2%, respectively, mainly due to infections or uncontrolled bleeding.

In responding patients, median duration of neutropenia in cycle 1 was 32 days (range 14–64), whereas median time to platelet recovery was 34 days (range 17–61).

Most frequent adverse events during induction one were infections, reported in 415 patients (80.9%). Of 415 patients, 138 had fever of unknown origin (33%), 215 had microbiologically documented infections (41.9%), and 62 had a clinically documented infection (25.1%). Most frequent infections were bacteremia, whereas 24% of patients developed grade > 2 adverse infectious events, mostly sepsis and pneumonia.

Severe mucositis (grade 3–4) was reported in 34 patients (6.5%), whereas a skin rash was reported in 113/513 patients (22%, grade 1–2 in 81 patients and grade 3–4 in 32).

After cycle I, among CR patients, 213 (71.7%) received a second cycle; specifically, 22 (7.4%) and 191 (63.3%) patients received the second induction or the first consolidation, respectively. Forty (13.6%) patients in CR discontinued treatment without proceeding to transplant, mainly due to prolonged cytopenia, whereas 44 (14.8%) underwent allo‐HSCT after cycle I.

After Cycle II, 77 patients proceeded to allo‐SCT, 48 did not receive further CPX‐351 therapy, and 88 received cycle III (8 Consolidation I and 80 Consolidation II); among them, 45 were allotransplanted.

Among the 216/513 (42.1%) patients who failed to achieve CR after induction I, 50 (23.2%) NR patients received induction II and 9 (4.1%) in PR received consolidation I. In the whole cohort, the total number of CPX‐351 cycles was one, two, or three in 241 (47%), 167 (32.6%), and 105 (20.5%) patients, respectively.

Among the 363 patients achieving at least a MLFS, the total number of CPX‐351 courses was 1, 2, or 3 in 91 (25.1%), 167 (46%), and 105 (28.9%) patients, respectively.

Multicolour flow cytometry (MFC) Minimal Residual Disease (MRD) assessment performed according to ELN 2022 recommendations was available in 92/217 responding patients (42%) and negative in 56 (61%) [22]. As previously reported, patients achieving MRD negativity had a significantly better survival if compared to MRD positive patients (Median OS 26 months vs. 16.4 months, *p* < 0.05, data not shown) [24]. Available data on MRD are provided in Supporting Information.

### 4.3 Impact of *NPM1, FLT3‐ITD, TP53*, and Cytogenetics on Response Probability

CR rate was significantly higher among *NPM1* mutated vs. non‐mutated patients (83.9% vs. 16.1%, respectively, *p* < 0.05, available *NPM1* MRD data are provided in Supporting Information) and among ELN 2017 favorable risk patients if compared to intermediate or high risk (77.8% vs. 71.1% vs. 48.5%, respectively, *p* < 0.05), whereas it was not affected by *FLT3*‐ITD mutations (45.8% vs. 58.5% among patients with or without *FLT3‐*ITD mutations, respectively, *p* = n.s.) or in the whole group of patients harboring a *TP53* mutations (61.2% and 57.1% among patients with or without a *TP53* mutations, respectively, *p* = n.s.). Patients with *TP53* mutations associated with a complex karyotype had a significantly lower CR rate if compared to all other patients (31.3% vs. 64.9%, for patients with or without *TP53* mutations in the context of a complex karyotype, respectively, *p* < 0.05). Age at diagnosis and previous HMA treatment did not impact CR probability. Multivariate analysis showed that favorable ELN 2017 risk score was the strongest, independent predictor of CR. Detailed CR analysis is provided in Table 2.

**TABLE 2:** Complete response probability.

		CR (%)	*p* (univariate)	*p* (multivariate)
Total patients		297/513 (57.9)	—	—
Age (Median 65.6)	< 60 years	118/199 (59.3)	0.324	—
	60–70 years	118/217 (54.4)		
	> 70 years	61/97 (62.9)		
Karyotype	Favorable Risk	5/5 (100)	0.087	0.478
	Intermediate	136/211 (64.5)		
	High Risk	156/297 (52.5)		
*NPM1*	Mutated	26/31 (83.9)	< 0.003	0.075
	Wild Type	271/482 (56.3)		
*FLT3‐ITD*	Positive	11/24 (45.8)	0.288	—
	Negative	286/489 (58.5)		
*TP53*	Mutated	28/49 (57.1)	0.436	—
	Wildtype	175/286 (61.2)		
ELN 2017	Favorable	21/27 (77.8)	< 0.001	< 0.05
	Intermediate	126/177 (71.2)		
	Unfavorable	150/309 (48.5)		
Type of AML	MRC‐AML	228/405 (56.3)	0.188	—
	t‐AML	69/108 (63.9)		
Previous HMA for MDS	Yes	48/84 (57.1)	0.904	—
	No	249/429 (58)		

Abbreviations: CR = complete remission; ELN = European LeukemiaNet; HMA = hypomethylating agents; MDS = myelodisplastic syndrome; WHO = World Health Organization.

### 4.4 Survival Analysis

After a median follow‐up of 23.66 months (95% CI 23.11–26.01), median OS was 16.23 months (95% CI 13.6–18.9, Figure S1), whereas 1‐year OS was 52.8%.

One‐year EFS was 41.6%, whereas median EFS was 9.3 months (95% CI 6.7–13.8, Figure S2). The majority of disease relapses were observed in the first 6 months after completion of treatment.

Compared to patients aged 60–70 or > 70, age younger than 60 years resulted in a significantly longer survival (median OS 21.4, 95% CI 13.2–29.7; 14.1, 95% CI 9.7–18.6; and 12.2, 95% CI 8.1–16.2, respectively, *p* < 0.03). At 2 years, 22.3% of patients aged 70 or more were alive, compared to 36.8% and 46.7% of those aged 60–70 or younger than 60, respectively. The rate of allo‐HSCT procedure was significantly higher among patients younger than 60 (*p* < 0.05).

### 4.5 Impact of *NPM1, FLT3‐ITD, TP53*, and Cytogenetics on Survival

Outcome was significantly superior in patients with *NPM1* mutations or belonging to the ELN 2017 favorable category. Indeed, median OS was not reached for these two categories (*p* < 0.05 for each), whereas it was 19.8 (IC 95% 16.1–23.4) and 11.7 months (IC 95% 8.9–14.5) for patients allocated to ELN 2017 intermediate or adverse categories (Figure S3). *TP53* mutations did not show a significant impact on OS unless a CK was concomitant (median survival 6.2 months, IC 95% 3.1–9.9; vs. 17.4 months, IC 95% 14.1–22.3 for *TP53* patients with or without a CK, respectively, *p* < 0.05, Figure S4). Furthermore, among patients with adverse risk cytogenetics, the presence of a mutated *TP53* correlated with a significantly worse OS (*p* < 0.05, Figure S4).

Previous HMA treatment for MDS did not significantly impact on OS (median OS 17.1, IC 95% 11–23.2, and 15.8, IC 95% 12.8–18.8, for patients who were previously exposed or not HMA, *p* = n.s.). Patients who were already exposed to HMA for MDS did not have different clinical characteristics if compared to other s‐AML patients (Table S1). Multivariate analysis of OS showed that ELN 2017 favorable‐risk was the strongest independent predictor of survival (*p* < 0.05) (Table 3).

**TABLE 3:** Overall survival analysis.

		Median OS months (95% CI)	*p* (univariate)	*p* (multivariate)
Total patients		16.23 (13.57–18.90)	—	—
Age (Median 65.6)	< 60 years	21.43 (13.20–29.66)	< 0.03	0.148
	60–70 years	14.13 (9.65–18.61)		
	> 70 years	12.2 (8.1–16.20)		
Karyotype	Favorable Risk	NR (−)	< 0.03	0.067
	Intermediate	17.89 (15.67–23.16)		
	High Risk	12.1 (9.13–16.41)		
*NPM1*	Mutated	24.6 (18.3‐NR)	< 0.03	0.093
	Wild Type	14.3 (11.8–16.8)		
*FLT3‐ITD*	Positive	12.8 (5.97–19.6)	0.908	—
	Negative	16.0 (13.28–18.66)		
*TP53*	Mutated	12.47 (9.29–16.26)	0.061	—
	Wildtype	18.6 (15.50–21.71)		
ELN 2017	Favorable	NR (−)	< 0.001	< 0.05
	Intermediate	19.78 (16.12–23.42)		
	Unfavorable	11.70 (8.88–14.52)		
Type of AML	MRC‐AML	15.97 (13.32–18.62)	0.869	—
	t‐AML	19.43 (9.49–29.41)		
Previous HMA for MDS	Yes	17.13 (11.04–23.22)	0.992	—
	No	15.78 (12.78–18.76)		
LANDMARK ANALYSIS				
Received ALLO‐HSCT	Yes	NR (−)	< 0.001	< 0.03
	No	16.57 (13.65–19.49)		
N. of CPX Courses (NO ALLO‐HSCT)	2 or less	13.30 (3.64–21.60)	0.039	—
	Three	20.37 (13.51–27.23)		
N. of CPX Courses (ALLO‐HSCT)	One	NR (−)	0.898	—
	More than one	34.93 (36.80‐NR)		

Abbreviations: allo‐HSCT = allogeneic hemopoietic stem cell transplantation; ELN = European LeukemiaNet; HMA = hypomethylating agents; MDS = myelodisplastic syndrome; OS = overall survival; WHO = World Health Organization.

EFS was significantly influenced by the presence of *NPM1* mutation (*p* < 0.05), favorable ELN score (*p* < 0.03), age younger than 60 years (*p* < 0.05), whereas it was not influenced by *FLT3*‐ITD or *TP53* mutations (unless a CK was present, *p* < 0.05) or previous HMA treatment.

Multivariate EFS analysis showed that ELN score was the only independent predictor of survival (*p* < 0.05).

### 4.6 Optimal Timing of Allo HSCT

Allo‐HSCT consolidation was performed in 230/513 patients (48.8%). The stem cell source, donor source, and conditioning intensity were comparable between patients receiving ASCT directly after cycle 1 or later (Supporting Information). After induction I, a total of 83 (36%) patients proceeded to Allo‐HSCT, with 44 (53%) being in CR and 39 (47%) in MLFS. Ninety received allo‐HSCT in CR after induction II, and 57 after consolidation I. Detailed timing of allo‐HSCT is provided in Figure 1. In order to assess the impact on OS of allo‐HSCT options, a landmark model was built, including patients alive and in CR at day 90, 120, and 150, starting from the day of CR assessment and considering HSCT as a time dependent covariate [23].

In competing risk analysis, considering death due to AML relapse as a competing event, non‐relapse mortality (NRM) among patients submitted to allo‐HSCT was 17% at 1 year and was not affected by the number of CPX‐351 courses received before transplantation, at any landmark considered. Among transplanted patients, no significant differences in terms of NRM were observed in patients receiving transplant directly after cycle 1 or later.

**FIGURE 2:**
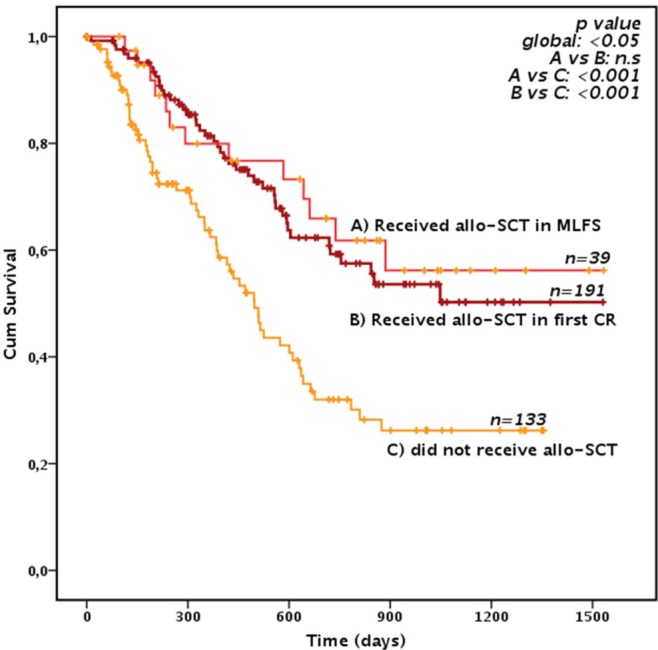
Overall survival according to transplant (landmark analysis). Figure 2 depicts the overall survival in a landmark analysis model of the 363 patients who achieved a morphological leukemia‐free status or a CR and were alive at day 90 (landmark) according to whether they received allogeneic stem cell transplantation consolidation, either in CR or in MLFS or did not receive transplant. [Color figure can be viewed at wileyonlinelibrary.com]

Furthermore, receiving an allo‐HSCT in first CR was the only variable related to a significantly longer OS in univariate analysis (median OS not reached and 16.3 months for patients receiving or not allo‐HSCT, respectively, *p* < 0.05, Figure 2). The advantage in terms of survival provided by allo‐SCT after each CPX‐351 cycle is shown in Figure S5.

Among patients in CR proceeding to allo‐HSCT, none of the other analyzed variables had an impact on survival, including *TP53* mutations and ELN risk group. Allo‐HSCT proved to be beneficial among intermediate and high‐risk ELN risk groups, but not among favorable‐risk patients (Figure S6). Among *TP53* mutated patients, the concomitant presence of a CK resulted in a significantly worse outcome also when allo‐HSCT was performed (Median OS 14.3 months, IC 95% 9.7–19.5, and not reached, for allo‐HSCT patients with or without a *TP53* mutation in the context of a CK, respectively, *p* < 0.05). Notably, 22.5% of *TP53* mutated patients with concomitant CK undergoing allo‐HSCT were alive at 2 years.

**FIGURE 3:**
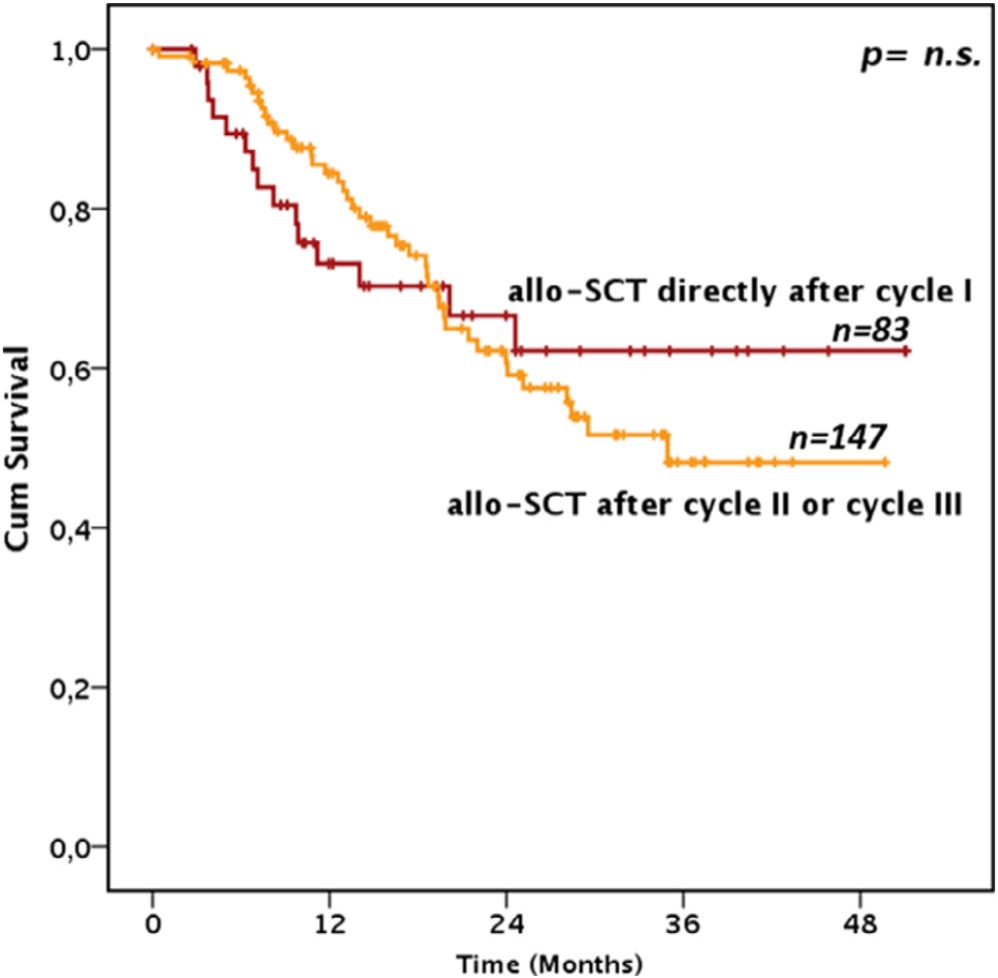
Survival of patients submitted to transplant based on the number of CPX‐351 courses received. Figure 3 depicts the overall survival in a landmark analysis model of the 230 patients who were alive and at least in a morphological leukemia‐free status at day 90 and received an allogeneic stem cell transplantation according to the number of CPX‐351 cycles received before the transplant. [Color figure can be viewed at wileyonlinelibrary.com]

After Induction I, receiving additional cycles before allo‐HSCT did not have any impact on OS (Median OS not reached, 35, IC 95% 28‐NR, and 28.4 months, IC 95% 26.3‐NR, in allo‐HSCT patients receiving 1, 2 or 3 CPX‐351 courses, respectively, *p* = n.s.). (*p* = n.s., Figure 3). Notably, relapse risk and transplant‐related mortality were superimposable in patients receiving one or more CPX‐351 courses before transplantation (*p* = n.s.). Furthermore, there was no difference in terms of OS, relapse risk or NRM in patients receiving allo‐HSCT after cycle I in a CR or MLFS status (Figure 2, *p* = n.s.).

**FIGURE 4:**
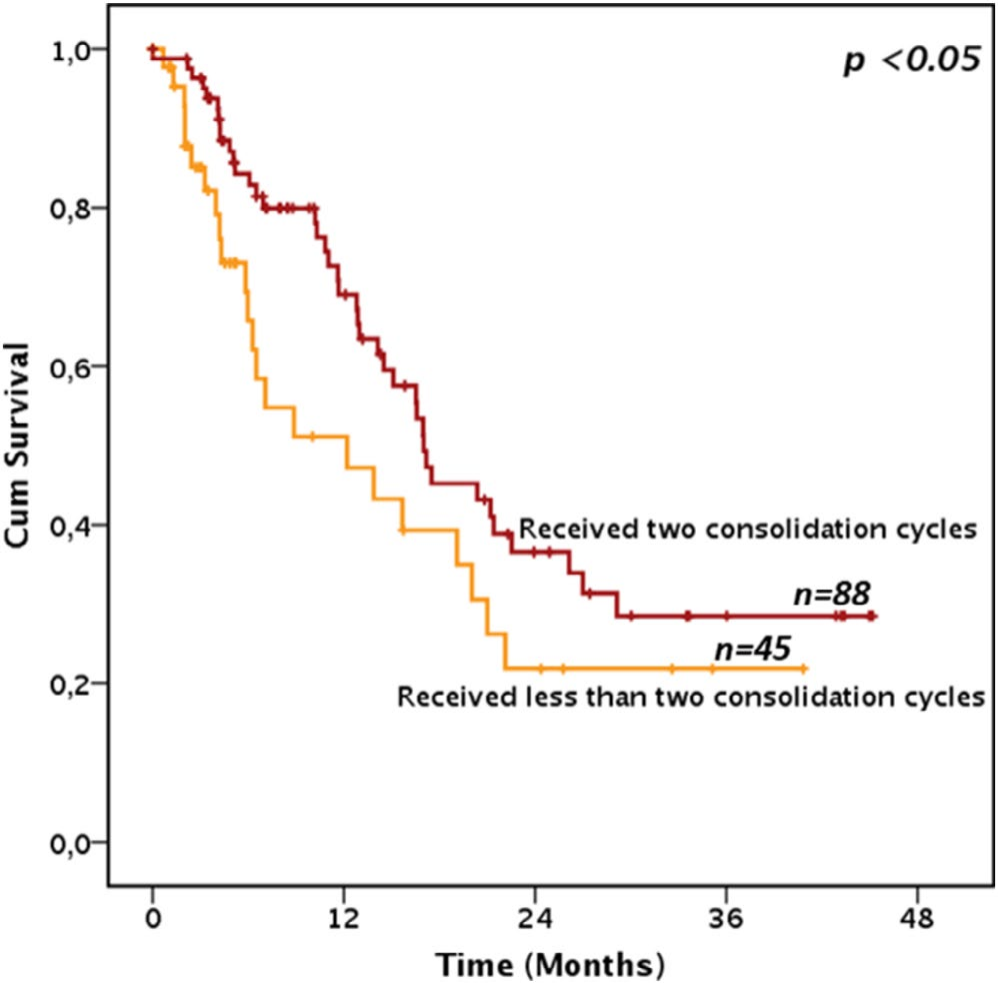
Survival according to number of consolidations in non‐transplanted patients. Figure 4 depicts the overall survival in a landmark analysis model of the 133 patients who were alive and at least in a morphological leukemia‐free status at day 90 and did not receive an allogeneic stem cell transplantation, according to the total number of CPX‐351 consolidation cycles received. [Color figure can be viewed at wileyonlinelibrary.com]

Indeed, receiving induction II (if needed) and consolidation I and II was associated with an OS advantage in non‐transplanted patients, as demonstrated by landmark analysis including patients in CR and alive at day 150 (median OS 20.37, IC 95% 13.51–27.2 and 13.3 months, IC 95% 3.6–21.6, in non‐transplanted patients receiving 3 vs. 2 or fewer CPX‐351 cycles, respectively, *p* < 0.05, Figure 4).

Multivariate OS analysis in the landmark model showed that receiving an allo‐HSCT was the strongest, independent predictor of survival (*p* < 0.03).

EFS analysis in the landmark analysis leads to superimposable results.

## 5 Discussion

The analysis of our real word series of patients, that at our knowledge is the largest and the only reporting detailed outcome after each CPX‐351 course, confirms that CPX‐351 can induce responses in a high proportion (70%) of s‐AML patients, with a manageable toxicity and prolonged OS [7, 8, 9, 10, 11, 12, 13]. The majority of response are quickly achieved after the first cycle. Overall, the low incidence of severe mucositis allowed allo‐HSCT to be delivered to a significant proportion of patients with a low rate of NRM. The probability of responding was not impacted by previous HMA exposure, confirming the results of the Italian Compassionate Use Program [9]. On the other hand, the efficacy of CPX‐351 in *TP53* mutated AML has always been a subject of controversy, with previous RWE generating conflicting results [9, 10, 11]. In our analysis, the presence of *TP53* mutations had a negative impact on CR achievement and survival only when combined with a CK. Those discrepancies may be explained by the heterogeneous sample size and methodology among the different studies. Consistently with previous experiences with CPX‐351, allo‐HSCT was the strongest predictor of long‐term survival [7, 8, 9, 10, 11, 12, 13].

In the Phase III 301 trial, patients who achieved a response in the CPX‐351 arm had a higher probability to proceed to allo‐HSCT and a significantly better post‐allo‐HSCT outcome compared to responding patients in the “3 + 7” arm [7]. Those results were confirmed in the subsequent extended follow‐up analysis. However, no data were provided concerning the optimal number of CPX‐351 cycles before allo‐HSCT [7, 14]. Since we did not observe any reduction in relapse risk with further CPX‐351 consolidation cycles before allo‐HSCT, our experience suggests that, in eligible patients, allo‐HSCT should be performed as soon as a CR is achieved in order to reduce the risk of treatment‐related toxicities. As most leukemic relapse was observed in the first months after CR, consolidation courses before transplant should be reserved for patients who delay or are not eligible for allo‐HSCT.

In this view, CPX‐351 showed superiority over conventional chemotherapy also in patients entering CR but not proceeding to allo‐HSCT, but again the optimal number of CPX‐351 cycles was not investigated and RWE studies did not address specifically this issue as well [8, 9, 10, 11, 15]. Our results suggest that among patients achieving a CR, receiving all the allowed CPX‐351 cycles results in a better outcome. It should be noted that in this study no patient received a maintenance treatment after completion of CPX‐351 treatment, as it was not yet approved in Italy.

In the QUAZAR trial, maintenance with oral azacytidine, given after achieving CR with intensive chemotherapy, led to a longer survival and delayed relapse in patients ineligible for allo‐HSCT [25]. However, in the QUAZAR trial, maintenance was tested after conventional “3 + 7” intensive treatment and proved to be more effective in patients who did not receive any consolidation [25]. Further studies on maintenance strategies following CPX‐351 are warranted, especially for those patients who achieve a CR with CPX‐351 but, due to hematological toxicity, are not able to proceed to further CPX‐351 treatment.

In the randomized phase III trial VIALE‐A, hypomethylating agents (HMA) in combination with the BCL‐2 inhibitor Venetoclax were superior to HMA alone in elderly AML patients ineligible for intensive chemotherapy [26]. More recently, there have been a number of studies reporting on the efficacy of HMA + Venetoclax also in “near‐fit or fit” patients, so that there is a certain proportion of patients who can be considered eligible for both CPX‐351 or HMA + Venetoclax, as first‐line treatment [27, 28, 29]. Data from comparative trials of CPX‐351 versus HMA plus Venetoclax or CPX‐351 with or without maintenance as first‐line treatment are not yet available [30, 31]. Therefore, we can only rely on the available indirect comparisons, mostly suggesting that the benefit from CPX‐351 may be higher in younger transplant‐eligible patients [30, 31]. A Phase Ib trial involving the use of a combination of reduced‐dose CPX‐351 plus Venetoclax for unfit patients is currently ongoing, but no results have been published so far (NCT04038437). Moreover, HMA + Venetoclax has been shown to be able to induce response and prolong survival also in a proportion of AML patients relapsed after or refractory to intensive chemotherapy; therefore, in the small proportion of patients who are eligible for CPX‐351 treatment but ineligible for allo‐HSCT, first‐line treatment with CPX‐351 followed by HMA‐Venetoclax as salvage treatment or to treat MRD may be a valid option [32, 33, 34].

Lastly, our large cohort allowed us to better analyze the impact of CPX‐351 in some AML subtypes which are usually underrepresented among s‐AML, such as *NPM1* or *FLT3*‐ITD mutated patients or favorable risk patients. Pre‐clinical cell line and ex vivo data demonstrated an increased uptake and cell‐killing with CPX‐351 if compared to conventional daunorubicine + cytarabine and suggested a synergistic effect of CPX‐351 and FLT3 inhibitors [35, 36, 37]. Previous RWE seemed to confirm a good activity, at least comparable to “3 + 7”, in *FLT3* mutated patients, however, the number of patients with *FLT3*‐ITD mutations were very few so that no significant conclusion could be drawn [9, 10, 11]. In our study, the presence of *FLT3*‐ITD did not significantly affect CR probability or survival in the 24 positive patients, suggesting that CPX‐351 may be used for s‐AML and t‐AML patients harboring these mutations. In *FLT3*‐ITD mutated patients, in the Phase III RATIFY trial, the addition of the multi‐kinase inhibitor midostaurin to conventional “3 + 7” allowed to achieve a significantly increased survival. However, in the RATIFY trial only younger, de novo AML patients were included [38]. In older patients two single arm trials confirmed the CR rate and the tolerability observed in the RATIFY trial, but no data on cytogenetics or efficacy on s‐AML patients were provided [39, 40]. Moreover, differently from our study, in those experience most patients had concomitant *NPM1* and *FLT3*‐ITD mutations, which may reduce the negative impact of *FLT3‐ITD* [38, 39, 40, 41]. Clinical trial combining CPX‐351 and FLT3‐targeting agents are ongoing but no results are available so far (NCT04128748). Therefore, no definitive conclusion on which is the better treatment in s‐AML and t‐AML with FLT3‐activating mutations can be drawn and, until comparative trial results are available, the decision should be individualized to the single patients considering cytogenetics, performance status and the presence of other mutations [37].

Even less data is available on the efficacy of CPX‐351 in s‐AML considered at low risk as per ELN classification or with a *NPM1* mutation [7, 8, 9, 10, 11]. In a recent Spanish study, CPX‐351 was retrospectively compared to intensive chemotherapy, demonstrating a similar CR/CRi rate and a slight advantage for OS in CPX‐351 treated patients [42]. In the French ALFA trial, the addition of Gemtuzumab‐Ozogamicin (GO) to 3 + 7 was shown to improve survival in low and, to a lesser extent, intermediate risk AML, as defined by cytogenetic analysis, but only de novo patients were included [43]. Subsequent experiences in *NPM1*‐mutated patients with conventional chemotherapy + GO failed to demonstrate an improved survival when compared to conventional chemotherapy alone [44]. In our experience, albeit with the limitations of small numbers, *NPM1*‐mutated patients and favorable risk patients in general showed very good outcomes with CPX‐351 treatment, regardless of being s‐AML. Given the good tolerability, CPX‐351 may be a valuable option for those rare subgroups of s‐AML patients. Combination trials involving CPX‐351 and GO are ongoing (NCT04915612).

## 6 Conclusions

In our large cohort of patients, we confirm the efficacy of CPX‐351 for the treatment of s‐AML and t‐AML. Albeit with the limitation of a retrospective study, it provides information that can be useful to optimize the use of this drug in terms of the number of courses to deliver and the appropriate time point for the allo‐HSCT option.

In eligible patients, allo‐HSCT should be performed as soon as a CR is achieved, whereas patients not proceeding to allo‐HSCT may still have a long survival if two consolidation courses are administered. In a future perspective, strategies of maintenance are to be considered to further improve the results of both transplanted and non‐transplanted patients.

Given the good tolerability, CPX‐351 may be used as a backbone instead of conventional 3 + 7 to investigate the addition of new, targeted drugs, and several studies are currently ongoing [17, 35, 36].

Finally, our study suggests that CPX‐351 may be beneficial also in rare subgroups of s‐AML such as *NPM1* mutated and ELN 2017 favorable risk patients or, to a lesser extent, in patients with mutated *FLT3‐ITD* [36].

## Author Contributions

**F.G., P.M., L.P., R.M.L., and, E.T.:** research conceptualization. **F.G., P.M., F.F., A.V., and, E.T.:** study design. **F.G., R.C., A.C., F.F., L.P., R.M.L., A.V., and, E.T.:** research overview. **M.P.M., F.L., F.G., F.P., C.F., D.C., P.C., M.B., S.M., M.B., M.B., M.F., S.G., V.M., A.L.P., N.F., C.P., F.G., P.Z., A.M., C.P., G.M., M.D., F.L., D.C., A.I., M.L., and, C.A.:** patient enrolment. **F.P., M.R., A.S., L.M., and, E.B.:** raw data collection. **C.V., M.C., S.G., C.R., G.M., and, I.L.:** data organization. **F.G., L.F., P.M., L.B., R.P., and, A.C.:** data analysis. **F.G., R.P., P.M., C.R., F.M., R.C., and, E.T.:** first draft. All authors approved the final version of the manuscript.

## Conflicts of Interest

Guolo: Jazz Pharmaceuticals Inc.: Consultancy; Fianchi: Jazz Pharmaceuticals Inc.: Honoraria; Sanofi: Honoraria; Bristol‐Myers Squibb: Honoraria. Martelli: AbbVie: Consultancy, Honoraria; Amgen: Consultancy, Honoraria; BMS: Consultancy, Honoraria; Laboratoires Delbert: Consultancy, Honoraria; Pfizer: Consultancy, Honoraria; Jazz Pharmaceuticals: Consultancy, Honoraria. Lussana: Bristol Myers Squibb: Membership on an entity's Board of Directors or advisory committees, Speakers Bureau; Incyte: Speakers Bureau; Clinigen: Membership on an entity's Board of Directors or advisory committees; Pfizer: Membership on an entity's Board of Directors or advisory committees, Speakers Bureau; AbbVie: Membership on an entity's Board of Directors or advisory committees; Amgen: Speakers Bureau. Breccia: Incyte: Honoraria; AbbVie: Honoraria; Novartis: Honoraria; AOP: Honoraria; Pfizer: Honoraria; BMS: Honoraria. Bocchia: Novartis: Honoraria; Incyte: Honoraria; BMS: Honoraria. Galimberti: AbbVie, Janssen, Novartis, Roche, Jazz, Astra Zeneca, Pfizer, Incyte: Speakers Bureau. Palmieri: Pfizer: Consultancy, Honoraria; Janssen: Consultancy, Honoraria; Jazz: Consultancy, Honoraria; AbbVie: Consultancy, Honoraria. Vetro: Jazz Pharmaceuticals: Honoraria; BMS: Honoraria; ABBVIE: Honoraria. Zappasodi: Amgen, Pfizer, AbbVie, Astellas: Honoraria. Borlenghi: AbbVie, BMS: Consultancy; Amgen, Incyte: Other: travel grants. Cerrano: Insight Novartis Servier AbbVie Janssen Jazz Astellas Italfarmaco: Honoraria. Papayannidis: AbbVie, Astellas, Servier, Menarini/Stemline, BMS, Pfizer, Amgen, Janssen, Incyte, Novartis: Honoraria; Pfizer, Astellas, Janssen, GSK, Blueprint, Jazz Pharmaceuticals, AbbVie, Novartis, Delbert Laboratoires: Membership on an entity's Board of Directors or advisory committees. Alati: AbbVie: Honoraria; Jazz: Honoraria. Fracchiolla: AbbVie, Jazz, Pfizer, Amgen: Other: travel grants; AbbVie, Jazz, Pfizer, Amgen: Speakers Bureau. Ferrara: ABBVIE: Honoraria. Venditti: Medac: Consultancy; Janssen: Consultancy, Honoraria, Other: travel support; AbbVie: Consultancy, Honoraria, Other: travel support; Jazz: Consultancy, Honoraria, Other: travel support; Amgen: Consultancy, Honoraria, Other: travel support; Pfizer: Consultancy, Honoraria, Other: travel support, Speakers Bureau; Novartis: Consultancy, Honoraria, Other: travel support. Pagano: Janssen: Honoraria; Pfizer: Honoraria; Gilead: Honoraria; Jazz: Honoraria; Novartis: Honoraria; Menarini: Honoraria; Moderna: Honoraria; AstraZeneca: Honoraria. The Authors state that they have no other relevant conflicts of interest to disclose.

## Supporting information


**Data S1:** Supporting Information.

## Data Availability

Deidentified individual participant data are available indefinitely upon request to the Corresponding Author at the address fabio.guolo@hsanmartino.it.
