# Supplementary material for: Real World Study on the Best CPX‐351 Treatment Duration and Timing for Allogeneic Stem Cell Transplantation
Source: Am J Hematol. 2025 Sep 24;100(12):2293–304. doi: 10.1002/ajh.70083 (PMC12603885; doi:10.1002/ajh.70083)
Supplement: Supplementary file 1 — Data S1: Supporting Information. [file AJH-100-2293-s001.docx]

**Supplementary Materials**

**ELN2022 risk classification**

For the 188/513 (37%) patients who had complete NGS data allowing correct risk stratification, the proportion of ELN 2022 risk groups were as follows: favourable 10/182 (6%), intermediate 34/182 (19%), unfavourable (79%). CR rate was not significantly different among different ELN 2022 group (8/10, 18/34 and 81/144 for favourable, intermediate and adverse risk group, respectively, p=n.s.) Overall survival was significantly longer for ELN 2022 favourable risk patients when compared to intermediate and adverse risk group whereas no significant difference was noted when comparing patients in intermediate or adverse risk group (median OS not reached vs 18.3 and 17.9 months, respectively, favourable vs intermediate p<0.05, favourable vs adverse p<0.05, intermediate vs adverse p=n.s.).

No significative differences in terms of Overall Survival were noted between patients belonging to the same prognostic group were comparing ELN 2022 and ELN Classification.

**Inpatient stay**

Data concerning hospitalization during cycle 1 were available in 174 patients (34%), median duration of inpatient stay was 31 days (range 24-91).

**MRD data**

MFC MRD performed according to ELN 2022 recommendations was available in 92/217 responding patients (42%) and negative in 56 (61%). Patients with negative MRD before ASCT had a survival advantage (Median OS not reached vs 23 months, p<0.05 for patients with negative or positive pre-transplant MRD, respectively).

Among the 92 patients with available MRD data, 58 received allo-HSCT in first CR, 31 directly after cycle 1 (53%) and 27 in a later phase (47%). MRD before transplant was negative in 39/58 patients receiving allo-HSCT (67%), with no significant differences between patients receiving allo-HSCT directly after cycle 1 compared to patients transplanted later (MRD negative in 21/31 and 18/27, respectively, p=n.s.)”

**Response and MRD in *NPM1* mutated patients**

Among the 31 *NPM1* mutated patients, 26 achieved a CR and 24/26 had available *NPM1* MRD data. After cycle 1, *NPM1* MRD was negative in 18/24 (75%) patients. The percentage of negative *NPM1* MRD negativity increased to 19/24 after cycle 2 (80%).”

**Outcome of patients with previous diagnosis of CMMoL**

Fifteen patients had a previous formal diagnosis of CMMoL. Median age in this subgroup was 69 years (range 62-74). After Cycle 1, CR rate was 8/15 (59%), increasing to 10/15 after cycle 2 (66%), not statistically different from the whole cohort. Median Overall Survival was 16.2 months, whereas 1 year OS was 44.1%, again non significantly different from the whole cohort.

**Stem Cell source and conditioning**

Among the 230 patients receiving allo-HSCT, complete data on HSCT characteristics were available in 183 patients (79%). Overall, 67 patients (37%) received a myeloablative conditioning, whereas 116 patients 863%) received a reduce intensity conditioning. Stem cell source was peripheral blood in the majority of cases (169/183, 92%), whereas donor source was matched unrelated donor in 39 patients (21%), HLA identical sibling donor in 69 (30%) and haploidentical donor in 75 patients (39%).

Among the 183 patients who had available HSCT data, 59 received transplant directly after cycle 1 (32%) and 124 in a later phase (68%).

When comparing patients receiving ASCT directly after cycle 1 or in a later phase, there was no significant difference in terms of conditioning intensity (intensive conditioning was performed in 22/59 and 47/124 patients, respectively). Similarly, there were no significant differences between patients receiving transplant after cycle 1 or later in terms of stem cell source (peripheral blood in 52/59 and 117/124, respectively) or donor source (MUD, HLA-ID and APLO in 12/59, 21/59 and 26/59, compared to 27/124, 48/124 and 49/124, respectively)”

**Outcome of transplanted patients according to ELN2017 risk score**

Among allo-HSCT patients, ELN risk group was favourable, intermediate or unfavourable in 8, 71 and 87, respectively, whereas patients included in the landmark model without receiving allo-HSCT in first CR were considered as favourable, intermediate or high risk in 13, 55 and 63 cases, respectively.

Allo-HSCT proved to be beneficial in unfavourable risk group (median OS 29.5 months and 11.63 months for patients transplanted or not in first CR, p<0.05), intermediate risk group (median OS not reached vs 16.97 months in patients receiving or not allo-HSCT in first CR, respectively, p<0.05), but not in the favourable risk group (median OS not reached both in patients receiving or not allo-HSCT in first CR, Fig. 6S).

**Supplementary Table T1S**

***Patient’s characteristics according to previous HMA treatment for MDS***

|  |  |  |  |  |
| --- | --- | --- | --- | --- |
| Total Patients |  | **Previous HMA for MDS** | **No Previous HMA treatment** | ***p*** |
| Age | <60 yrs | 27 (32.1%) | 172 (40.1%) | 0.094 |
|  | 60-70 yrs | 34 (40.5%) | 183 (42.7%) |  |
|  | >70 yrs | 23 (27.4%) | 74 (17.2%) |  |
| *TP53* | Mutated | 4 (7.5%) | 46 (16.3%) | 0.139 |
|  | Wildtype | 46 (92.5) | 236 (83.7%) |  |
| ELN 2017 | Favorable | 6 (7.1%) | 21 (4.9%) | 0.764 |
|  | Intermediate | 24 (28.6%) | 153 (35.7%) |  |
|  | Unfavorable | 54 (64.3%) | 255 (59.4%) |  |

**Table Legend: HMA= Hypomethylating Agents; MDS= Myelodisplastic Syndrome; ELN= European LeukemiaNet**

**Figure 1S: Overall Survival in the whole cohort**


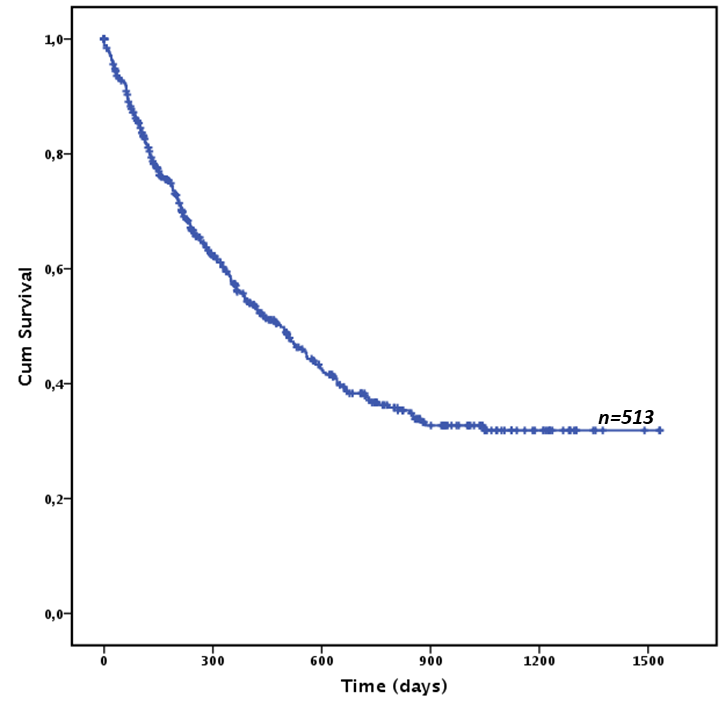


**Figure 2S: Relapse Free Survival in the whole cohort (censored at transplant)**


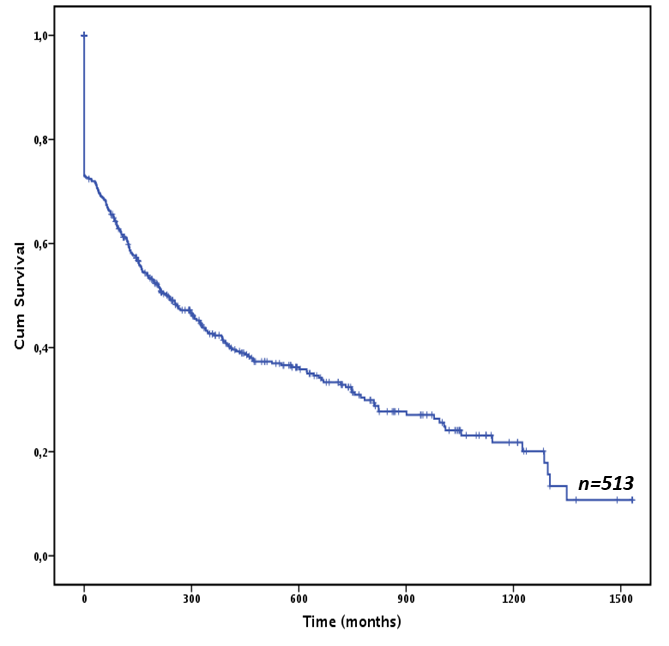


**Figure 3S: Overall Survival According to European LeukemiaNET 2017 risk score in the whole cohort**

**
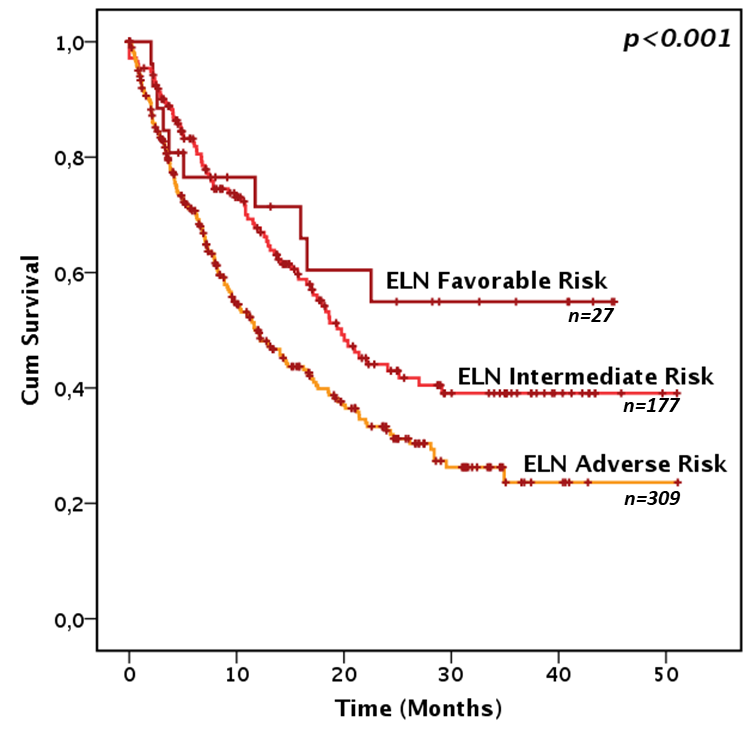
**

**Figure 4S: Overall Survival According to TP53 mutational status and complex karyotype in the whole cohort**


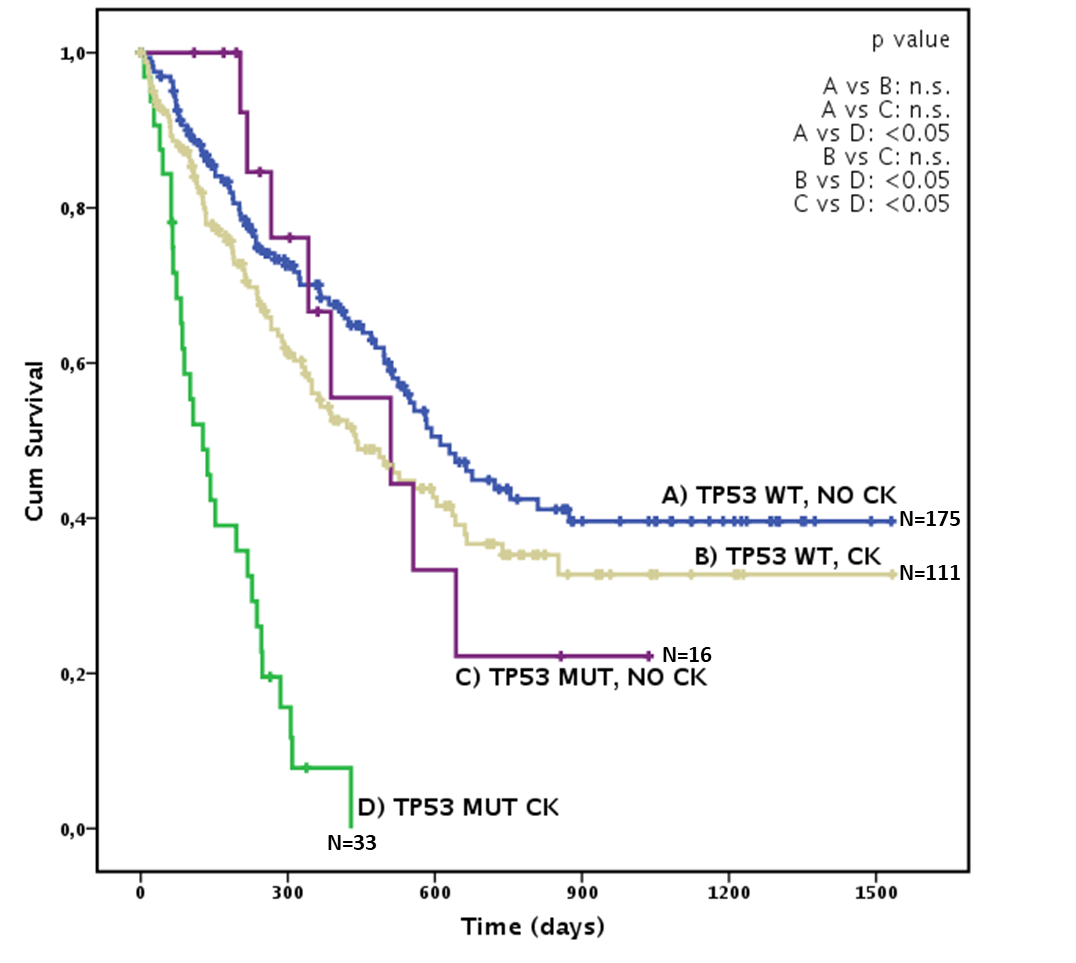


**Figure 5S: Overall Survival in CR patients after each CPX-351 course according to receiving or not a allogeneic stem cell transplantation.**


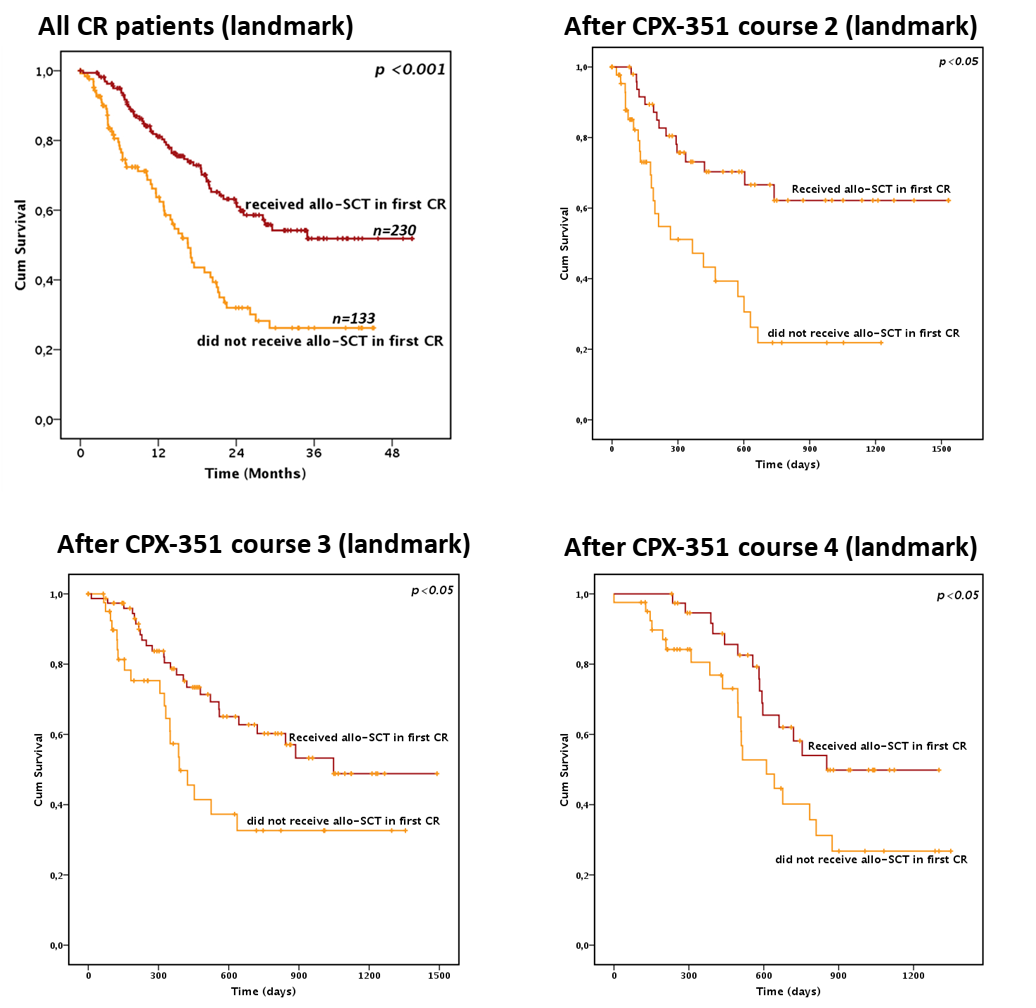


**Figure 6S: Overall Survival according to allo-HSCT in different ELN 2017 risk groups.**

**6SA: favourable risk; 6SB: intermediate risk; 6SC: unfavourable risk.**

**
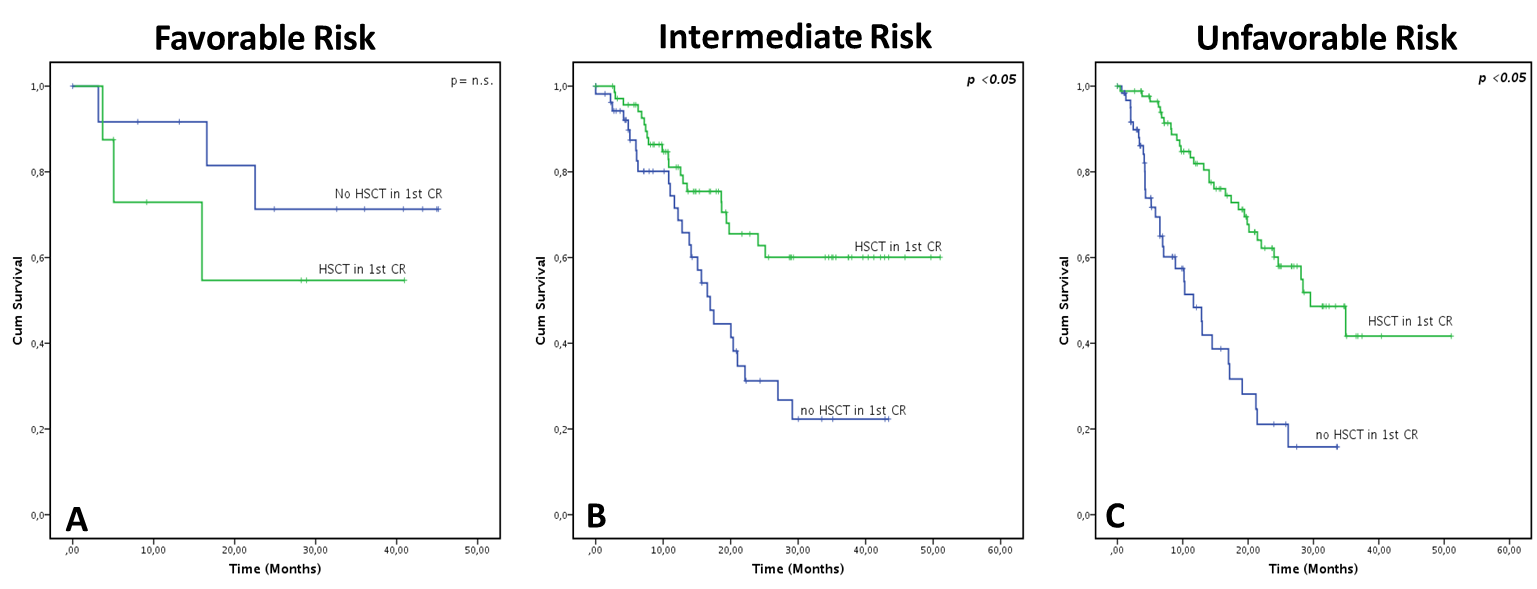
**
